# A hybrid ConvNeXt–BiLSTM framework for robust scene text recognition

**DOI:** 10.1038/s41598-026-50234-6

**Published:** 2026-05-13

**Authors:** Alshefaa Khattab, Marwa Elpeltagy, Farida Youness, Ahmed Elshafei, Ahmed Y. Khedr

**Affiliations:** 1 Department of Systems and Computers, Al-Azhar University, Cairo, Egypt; 2 Department of Communications and Computers, Higher Institute of Engineering, AL-Shorouk, Cairo, Egypt

**Keywords:** Convolutional network next, Scene text recognition, Label smoothing, Focal loss, Deep learning, Engineering, Mathematics and computing

## Abstract

Scene Text Recognition (STR) is a fundamental computer vision task with broad applications in autonomous navigation, document digitization, and assistive technologies. However, traditional STR models often rely heavily on large synthetic datasets due to the scarcity of annotated real-world data, which limits their generalization in complex environments. To address this challenge, this study proposes a ConvNeXt-based deep learning framework that integrates Convolutional Network Next (ConvNeXt) for robust feature extraction with Bidirectional Long Short-Term Memory (BiLSTM) networks for effective sequence modeling. The framework incorporates label smoothing and focal loss to enhance training stability and alleviate class imbalance and overconfidence issues. Training is conducted in two stages: pre-training on synthetic datasets (MJSynth and SynthText) followed by fine-tuning on diverse real-world datasets, including IC13, IC15, RCTW, ArT, LSVT, MLT19, ReCTS, COCO-Text, Uber-Text, TextOCR, OpenVINO, and a subset of Union14M-L. Experimental results demonstrate that the proposed model achieves an average accuracy of 94.71% over six standard STR benchmarks (IIIT5k, SVT, IC13, IC15, SVTP, and CUTE80) when trained on both synthetic and real datasets, surpassing the 89.1% accuracy achieved using synthetic data alone on the same benchmarks, and outperforming state-of-the-art methods trained under comparable data conditions. The integration of ConvNeXt, BiLSTM, advanced loss functions, and heterogeneous datasets substantially improve STR performance, particularly under challenging conditions involving irregular text layouts, multilingual content, and complex backgrounds. Furthermore, the complete recognition pipeline achieves 20.3 M parameters, 1.9 GFLOPs, and an inference latency of 2.638 ms per image, demonstrating the practical suitability of the proposed framework for real-time deployment.

## Introduction

Scene Text Recognition (STR) has emerged as a pivotal task in computer vision, aiming to automatically extract textual information from natural scene images. It plays a vital role in numerous applications, including visual question answering (VQA), autonomous driving, augmented reality, and intelligent document analysis^1^. Unlike traditional Optical Character Recognition (OCR) systems that function effectively in controlled environments, STR operates in complex real-world settings characterized by diverse backgrounds, irregular text shapes, various font styles, low resolutions, occlusions, and perspective distortions^2^. The rapid progress of deep learning has significantly transformed STR research. Convolutional Neural Networks (CNNs) have become the foundation for hierarchical feature extraction from text regions^3^. At the same time, Recurrent Neural Networks (RNNs) and Long Short-Term Memory (LSTM) models effectively capture sequential dependencies in character sequences^4^. Notably, these fundamental architectures (CNNs, RNNs/LSTMs) have demonstrated remarkable success across diverse computer vision applications, including medical image analysis and enhancement^5–7^, intelligent transportation systems^8,9^, and multimedia content verification^10^, underscoring their versatility. Moreover, attention mechanisms have recently been incorporated to allow models to selectively focus on salient text regions, thereby improving recognition accuracy in noisy and distorted scenes^11^. The challenge of designing robust feature representations capable of resisting input-level perturbations—whether arising from real-world degradation or adversarial conditions—has been identified as a unifying theme across visual recognition tasks^12^. This perspective reinforces the motivation for the multi-scale normalization and attention mechanisms proposed in the present work, which address perturbation-like challenges in the form of background clutter, geometric distortion, and non-uniform illumination inherent to scene text.

A persistent limitation in STR development has been the lack of large-scale, annotated real-world datasets. Earlier methods predominantly relied on synthetic datasets such as MJSynth and SynthText to learn generalizable representations^13^. However, the emergence of large, labeled real-world datasets has enabled researchers to combine synthetic and real data to enhance generalization and robustness in complex, unconstrained environments^14^. In this work, we propose a ConvNeXt-based architecture for feature extraction, leveraging its modernized convolutional design that achieves performance comparable to Vision Transformers while maintaining computational efficiency^15^. The extracted features are further refined using BiLSTM networks for effective sequence modeling, facilitating robust recognition across irregular, multi-oriented, and multilingual text instances. To further improve training stability and model generalization, Label Smoothing Regularization (LSR)^16^ and Focal Loss^17^ are integrated into the optimization process, reducing overconfidence and addressing class imbalance during training. The primary contributions of the paper are as follows:

- A ConvNeXt-based feature extractor is introduced for scene text recognition, enabling more efficient and robust hierarchical feature representation compared to conventional hybrid architectures.
- Bidirectional Long Short-Term Memory (BiLSTM) networks are employed to enhance sequence modeling and improve recognition accuracy.
- The training strategy incorporates both large-scale synthetic datasets and newly available real-world labeled datasets, effectively addressing the generalization gap in real-world STR scenarios.
- Advanced loss functions, specifically Label Smoothing and Focal Loss, are integrated into the optimization process to handle class imbalance and overconfidence, leading to improved model robustness.
- Extensive experiments across multiple benchmarks establish new performance baselines, demonstrating the effectiveness of ConvNeXt feature extraction, dataset diversity, and optimized training strategies.

Recent years have witnessed remarkable progress in STR, driven by advancements in deep learning. However, the lack of consistent benchmarking has hindered fair comparisons across models. Baek et al.^11^ addressed this by introducing a unified four-stage STR framework and standardized evaluation protocols, enabling systematic analysis of recognition accuracy, computational efficiency, and memory use. Similarly, Wan et al.^18^ investigated vocabulary dependence in STR, revealing that attention-based models struggle with out-of-vocabulary words, while segmentation-based decoders generalize better. They further proposed a mutual learning strategy that allows both paradigms to exchange knowledge for improved performance.

Transformer-based models have gained traction in STR. Raisi et al.^19^ enhanced spatial feature preservation using a two-dimensional positional encoder, outperforming state-of-the-art methods on irregular-text datasets. Du et al.^20^ explored temporal convolutional encoders for capturing long-term dependencies, while Li et al.^21^ proposed a Two-Dimensional Multi-Scale Perceptive Context (TDMSPC) module to improve multi-scale feature learning. Luo et al.^22^ introduced a learnable text image augmentation framework that jointly optimizes augmentation and recognition, boosting performance across benchmarks.

The creation of large real-world datasets has further strengthened STR research. Singh et al.^23^ released TextOCR, featuring 900,000 annotated words, which significantly improved OCR generalization. Meanwhile, Qiao et al.^24^ refined attention mechanisms using Gaussian Constrained Attention Networks, achieving more focused and stable attention distributions. Cui et al.^25^ proposed the RCEED framework to enhance encoder-decoder feature interaction, improving contextual understanding during recognition.

To improve interpretability, Liu et al.^26^ designed a Character-Context Decoupling (CCD) model that separates visual and linguistic cues, enabling robust sequential reasoning. Slossberg et al.^27^ addressed model overconfidence through word-level calibration techniques, reducing calibration error by a factor of seven. Zhong et al.^28^ introduced SGBANet, combining Semantic GANs and Balanced Attention for semantically guided feature generation. Similarly, Xue et al.^29^ developed I2C2W, a two-stage image-to-character and character-to-word model resilient to geometric and photometric distortions.

Recent multimodal and language-informed STR frameworks have further improved contextual understanding. Wang et al.^30^ proposed CLIP-OCR, integrating linguistic and visual representations using symmetrical distillation. Zhang et al.^31^introduced the Linguistic Perception Vision (LPV) model, leveraging language reconstruction to correct linguistic deviation, while Xu et al.^32^ proposed the One Token Recognizer (OTE) for global semantic encoding and flexible decoding. Zhao et al.^33^ proposed CLIP4STR, an effective scene text recognition (STR) framework that leverages the pre-trained image and text encoders of CLIP. The approach integrates two complementary encoder–decoder branches: a visual branch and a cross-modal branch. The visual branch produces an initial recognition result based solely on visual features, while the cross-modal branch refines this output by explicitly reducing the semantic discrepancy between visual representations and textual embeddings. Du et al.^34^ investigated the role of autoregressive (AR) decoding in STR and demonstrated that AR decoders effectively model linguistic context while simultaneously guiding visual feature perception. Building on this analysis, they proposed the Context Perception Parallel Decoder (CPPD), which predicts character sequences in a single parallel inference step through three specialized components: character counting, character ordering, and character prediction. Yang et al.^35^ presented a parallel and iterative decoding framework based on an easy-first decoding strategy, reformulating scene text recognition as an image-conditioned text generation problem. By incorporating a discrete diffusion mechanism, the proposed method efficiently exploits bidirectional contextual dependencies. Extensive experiments on standard benchmarks for both Chinese and English texts demonstrate its superior recognition performance. Finally, Du et al.^36^ advanced task-specific augmentation through adaptive fiducial-point learning, jointly optimizing augmentation and recognition.

A comparative summary of these studies and their methodologies is presented in Table 1, which outlines the key architecture, techniques, datasets, and performance outcomes discussed in this section.

**Table 1:** Literature study on existing methodology.

No	Author & Year	Methodology	Dataset	Performance
1	Beak et al.^11^, 2019	TRBA	ITTT, SVT, IC13_857_, IC13_1015_, IC15_1811_, IC15_2077_, SVTP, CUTE	Accuracy (87.9, 87.5, 93.6, 92.3, 77.6, 71.8, 79.2, 74.0)
2	Fang et al.^14^, 2021	ABINet-LV†	ITTT, SVT, IC13_857_, IC15_1811_, SVTP, CUTE	Accuracy (96.3, 93.0, 97.0, 85.0, 88.5, 89.2)
3	Yu et al.^37^, 2020	SRN	ITTT, SVT, IC13_857_, IC15_1811_, SVTP, CUTE	Accuracy (94.8, 91.5, 95.5, 82.7, 85.1, 87.8)
4	Qiao et al.^38^, 2020	SEED	ITTT, SVT, IC13_1015_, IC15_1811_, SVTP, CUTE	Accuracy (93.8, 89.6, 92.8, 80.0, 81.4, 83.6)
5	Wan et al.^39^, 2020	TextScanner	ITTT, SVT, IC13_1015_, IC15_1811_, SVTP, CUTE	Accuracy (95.7, 92.7,94.9,83.5,84.8, 91.6)
6	Litman et al.^40^ , 2020	SCATTER	ITTT, SVT, IC13_1015_, IC15_2077_, SVTP, CUTE	Accuracy (93.7, 92.7, 93.9, 82.2, 86.9, 87.5)
7	Yue et al.^41^, 2020	RobustScanner	ITTT, SVT, IC13_1015_, IC15_2077_, SVTP, CUTE	Accuracy (95.3, 88.1, 94.8, 77.1, 79.5, 90.3)
8	Yan et al.^42^ 2021	Base2D PREN2D	ITTT, SVT, IC13_857_, IC15_1811_, SVTP, CUTE	Accuracy (95.4, 93.4, 95.9, 81.9, 86.0, 89.9) Accuracy (95.6, 94.0, 96.4, 83.0, 87.6, 91.7)
9	Atienza.,^43^, 2021	ViTSTR	ITTT, SVT, IC13_857_, IC13_1015_, IC15_1811_, IC15_2077_, SVTP, CUTE	Accuracy (88.4, 87.7, 93.2, 92.4, 78.5, 72.6, 81.3)
10	Wang et al.^44^, 2021	VisionLAN	ITTT, SVT, IC13_857_, IC15_1811_, SVTP, CUTE	Accuracy (95.8, 91.7, 95.7, 83.7, 86.0, 88.5)
11	Cai et al.^45^, 2021	STN-CSTR	ITTT, SVT, IC13_857_, IC13_1015_, IC15_1811_, IC15_2077_, SVTP	Accuracy (94.2, 92.3, 96.3, 94.1,86.1, 82.0, 86.2)
12	Qiao et al.^46^, 2021	PIMNet	ITTT, SVT, IC13_857_, IC13_1015_, IC15_1811_, IC15_2077_, SVTP, CUTE	Accuracy (95.2, 91.2, 95.2,93.4, 83.5,81.0,84.3, 84.4)
13	Zhang et al.^47^, 2022	ABINet-Vision-ConCLR	ITTT, SVT, IC13_857_, IC15_1811_, SVTP, CUTE	Accuracy (95.7, 92.1, 95.9, 84.4, 85.7, 89.2)
14	Selvam et al.^48^, 2022	OATSA	ITTT, SVT, IC13_1015_, IC15_2077_, SVTP, CUTE	Accuracy (97.7, 96.6, 98.0, 88.2, 90.6, 91.3)
15	Du et al.^49^, 2022	SVTR	ITTT, SVT, IC13_857_, IC15_1811_, SVTP, CUTE	Accuracy (96.3, 91.7, 97.2, 86.6, 88.4, 95.1)
16	Wang et al.^50^,2022	MGP-STR	ITTT, SVT, IC13_857_, IC15_1811_, SVTP, CUTE	Accuracy (96.40, 94.74, 97.32, 87.24, 91.01, 90.28)
17	Wang et al.^30^ ,2023	CLIP-OCR	ITTT, SVT, IC13_857_, IC15_1811_, SVTP, CUTE	Accuracy (97.3, 94.7, 97.7, 87.2, 89.9, 93.1)
18	Zhang et al.^31^,2023	LPV-Base	ITTT, SVT, IC13_857_, IC15_1811_, SVTP, CUTE	Accuracy (97.3, 94.6, 97.6, 87.5, 90.9, 94.8)
19	Xu et al.^32^, 2024	OTEA / ViT-B	ITTT, SVT, IC13_1015_, IC15_2077_, SVTP, CUTE	Accuracy (96.4, 95.5, 97.9, 86.8, 91.9, 90.3)
20	Zhao et al.^3,3^,2024	CLIP4STR-B	ITTT, SVT, IC13_1015_, IC15_1811_, IC15_2077_, SVTP, CUTE	Accuracy (97.7, 95.2, 96.1, 87.6, 84.2, 91.3, 95.5)
21	Du et al.^34^,2025	SVTR-T-CPPD	ITTT, SVT, IC13_857_, IC15_1811_, SVTP, CUTE	Accuracy (96.6, 94.4, 97.1, 86.6, 88.5, 90.3)
22	Yang et al.^35^,2025	IPAD	ITTT, SVT, IC13_857_, IC13_1015_, IC15_1811_, IC15_2077_, SVTP, CUTE	Accuracy (96.8, 94.3, 97.0, 95.6, 86.7, 83.0, 89.3)

Collectively, these works underscore the evolution of STR from purely convolutional approaches toward hybrid models that integrate transformers, attention mechanisms, and linguistic priors. This trend towards robust, context-aware vision systems is mirrored in adjacent research domains.

## Methods and materials

### Materials

#### Synthetic datasets for training

**Fig. 1:**
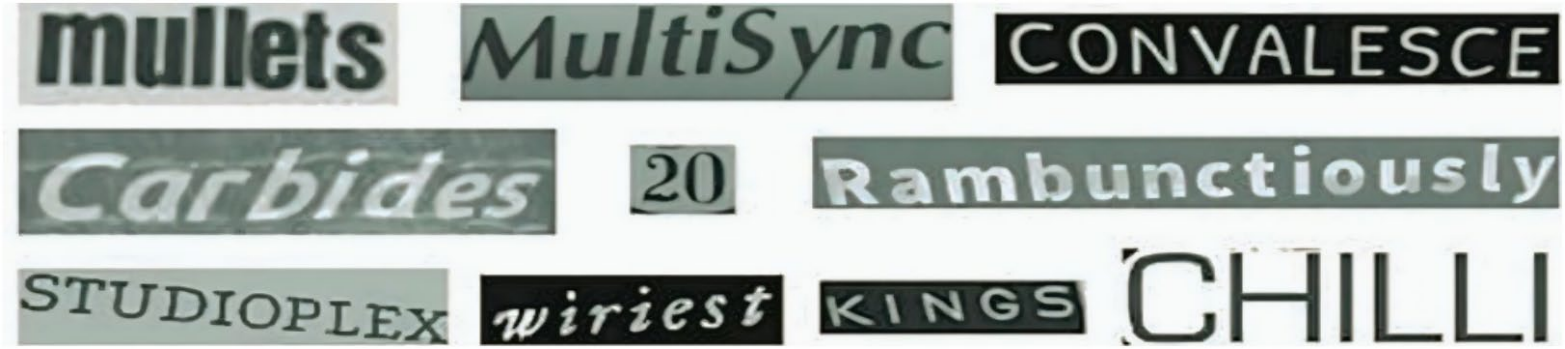
Samples of SynthText(ST) and MJSynth (MJ) datasets.

There are two important synthetic datasets: MJSynth (MJ)^51^ and SynthText (ST)^52^, which are listed in Table 2. Figure 1 illustrates samples of the MJ and ST datasets.

**Table 2:** Synthetic datasets for str training.

Dataset	Description	Size
MJSynth (MJ)	Specifically designed for STR, this dataset contains 9-million-word boxes. Each word is created from a 90,000-word English lexicon and uses over 1,400 Google Fonts	9 M word boxes
SynthText(ST)	The text was initially created for STR and is displayed on images of natural scenes. The chopped text portions serve as the training data for STR	7 M word boxes

#### Labeled real datasets for training

An increasing number of irregular text samples have been accumulated in recent years. Adding these texts to training datasets can improve STR models’ resilience. Several genuinely labeled datasets, including ITTT, IC13, IC15, RCTW, ArT^53^, LSVT, MLT19, ReCTS, COCO, Uber, TextOCR, OpenVINO, and a portion of Union14M-L, have been made available through ICDAR competitions. The collection of real-labeled datasets has grown because of these biennial challenges. A summary of the datasets published every two years can be seen in Table 3:

**Table 3:** Labeled real datasets for STR train.

Year	Dataset	Description
2013	IIIT	Collected from Google image searches, containing text from billboards and posters
2013	IC13	Created for ICDAR 2013, primarily consisting of horizontal text samples
2015	IC15	Captured using Google Glass, containing blurry, perspective, and low-resolution text samples due to motion effects
2017	COCO-Text (COCO)^54^	Derived from the MS COCO dataset^55^, featuring occluded and low-resolution text samples
2017	RCTW^56^	Developed for the Reading Chinese Text in the Wild competition, featuring many Chinese text samples
2017	Uber-Text (Uber)	Collected from Bing Maps Streetside and primarily contains house numbers, with some text appearing on signboards
2019	ArT	Designed for recognizing arbitrary-shaped text, including perspective and curved texts. It integrates Total Text^57^ and CTW1500^58^, which also feature rotated and curved texts
2019	LSVT	A large-scale street view text dataset collected in China, predominantly featuring Chinese text samples
2019	MLT19	Focuses on recognizing multilingual text across seven languages: Arabic, Latin, Chinese, Japanese, Korean, Bangla, and Hindi
2019	ReCTS	Created for the Reading Chinese Text on Signboards competition, showcasing irregular text layouts and unique fonts
2021	TextOCR	It is a large-scale dataset for STR, comprising approximately 900,000 high-quality word-level annotations extracted from approximately 28,000 images in the Open Images dataset. It is designed to support robust training and evaluation of STR models across diverse scripts, orientations, and real-world imaging conditions
2021	OpenVINO	refers to the text annotation for the Open Images V5 dataset, which is the largest publicly available, manually created text annotation dataset, designed for training high-quality deep learning models for text spotting tasks like OCR
2023	Union14M-L	Comprising over four million labelled images from diverse real-world scenarios, incorporates a challenge-driven benchmark of six subsets (409,393 images) specifically designed to address complex STR cases, including curved, multi-oriented, and artistic text

STR models can be trained on a wide range of text features, including perspective distortions, different resolutions, multilingual scripts, and intricate layouts, by incorporating these actual labeled datasets into the training dataset. Increased model performance and adaptation to real-world situations are fostered by this diversity. The methodology outlined in Baek et al.^59^ was used to identify the preprocessing steps applied to the labeled real datasets.

To ensure consistency and avoid bias, a unified harmonization pipeline was applied to all 12 training datasets: (1) word-level crops were resized to 32 × 100 in accordance with the standard STR protocol Baek et al.^59^; (2) vocabulary filtering was used, keeping only 36-class alphanumeric samples (a–z, 0–9), excluding non-Latin characters from multilingual datasets while keeping their Latin-script samples for visual diversity; (3) class-balanced batch sampling was used, regardless of the size of each dataset; and (4) all six evaluation benchmarks were strictly excluded from the training pool to prevent data leakage.

#### Real datasets for evaluation

As indicated in Table 4, six real datasets are used to evaluate the efficacy of STR models. These datasets are separated into regular and irregular sets based on text orientation and arrangement.

**Table 4:** Real datasets for evaluation.

Dataset	Description	Category	Training Images	Evaluation Images
Street View Text (SVT)^60^	Collected from Google Street View, this dataset features text from street scenes	Regular	257	647
IIIT5K-Words (IIIT)^61^	Compiled from Google Image searches, including text from billboards and movie posters	Regular	2,000	3000
ICDAR2013 (IC13)^62^	Collect from the ICDAR 2013 Competition. Designed for Robust Reading, mainly horizontal text	Regular	848	1,015
ICDAR2015 (IC15)^63^	Captured using Google Glass, this dataset contains perspective and blurry text images	Irregular	4,468	2,077
SVT Perspective (SP)^64^	Collected from Google Street View, focuses on **perspective text**	Irregular	–	645
CUTE80 (CT)^65^	Collected from Digital Cameras & Internet, focused on curved text	Irregular	–	288

### Methods

**Fig. 2:**
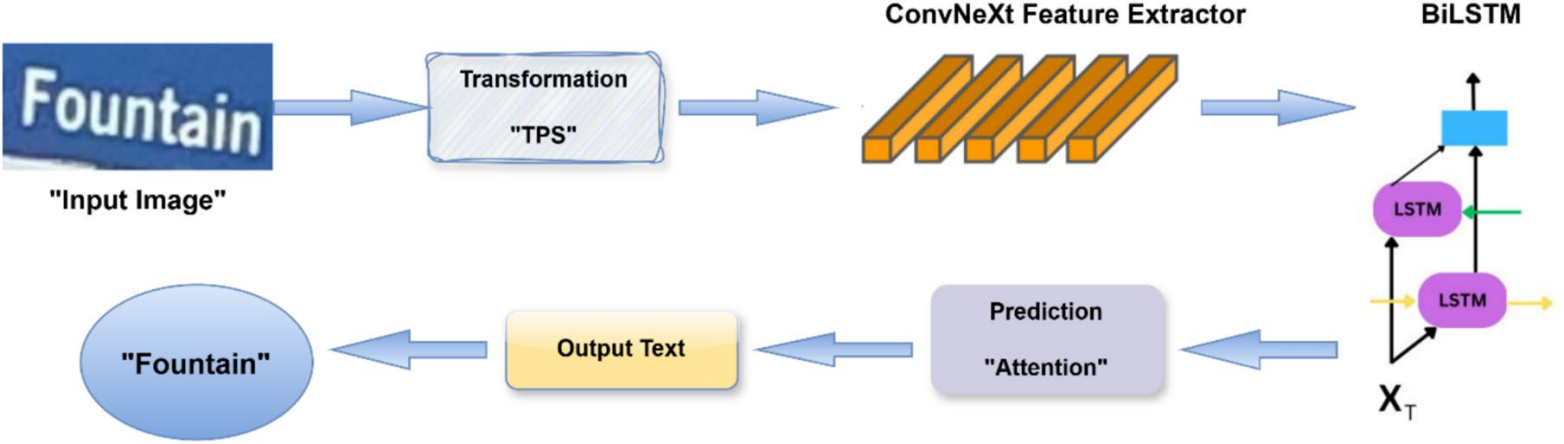
Pipeline of the proposed STR system using TPS, ConvNeXt Model, BiLSTM, and prediction stage for text recognition.

The proposed ConvNeXt-based Feature Extraction Model for STR is presented in this section. As depicted in Fig. 2, the overall methodology is organized into four main stages: transformation, feature extraction, sequence modeling, and prediction.

**Fig. 3:**
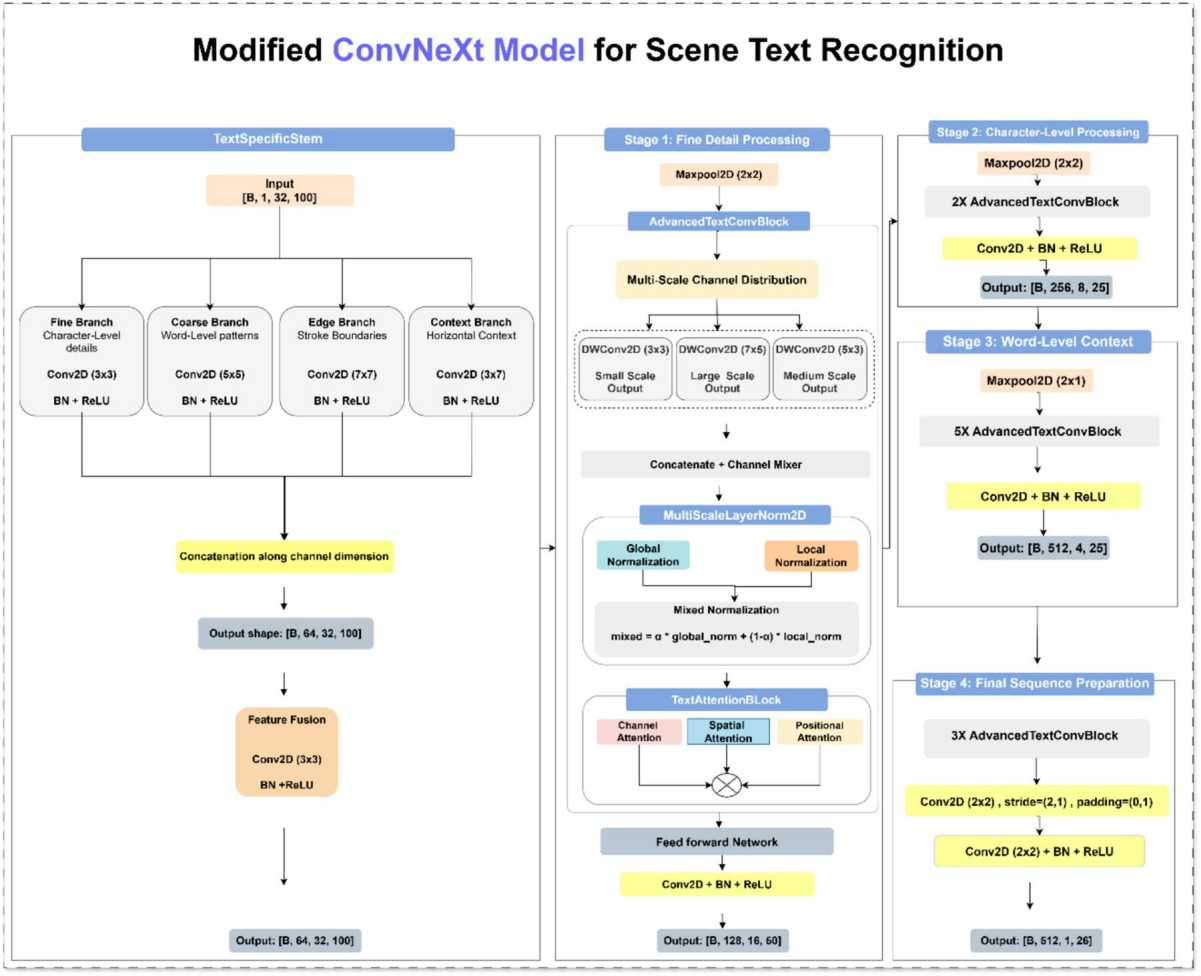
Architecture of the integrated ConvNeXt framework.

The detailed architecture of the integrated ConvNeXt framework, specifically tailored for STR applications, is illustrated in Fig. 3. To effectively tackle the inherent challenges of STR—such as irregular text shapes, multilingual scripts, background complexity, and varying orientations—the ConvNeXt backbone has been carefully refined and optimized to ensure enhanced robustness, computational efficiency, and strong generalization across diverse real-world scenarios.

#### Transformation stage

The Transformation Stage is responsible for correcting geometric distortions that commonly occur in natural scene images. Text captured in real-world settings often appears curved, rotated, or skewed due to camera angles, perspective shifts, or environmental factors. To address these challenges, a Thin-Plate Spline (TPS) Spatial Transformer Network is employed. The TPS module learns a flexible and non-linear mapping function that warps the input image into a rectified form, ensuring consistent text alignment. This geometric normalization enhances the model’s ability to focus on textual features rather than distortions. Consequently, the transformation stage significantly improves robustness and recognition accuracy, providing well-aligned inputs for subsequent feature extraction.

#### Feature extraction stage

Once the input images are geometrically normalized, the next stage focuses on extracting high-level discriminative features that effectively represent textual patterns. In this work, the ConvNeXt (Convolutional Network Next) architecture is adopted as the backbone for feature extraction. ConvNeXt is a modernized convolutional neural network that combines the efficiency and inductive biases of traditional CNNs with architectural principles inspired by Transformers. It integrates several key enhancements, including large-kernel convolutions, inverted bottlenecks, and layer normalization, enabling the model to capture long-range spatial dependencies while maintaining computational efficiency.

Although Transformer-based architectures, like Vision Transformer (ViT) and Swin Transformer, have proven to perform well on general visual recognition tasks, their direct application to word-level scene text recognition (STR) raises efficiency concerns because this field typically uses a relatively small input resolution (32 × 100). For example, Swin Transformer versions range from 29 to 88 M parameters depending on the model scale^66^, but ViTSTR-Base, which adapts the ViT architecture for STR, requires about 85.8 M parameters^43^. ConvNeXt, on the other hand, incorporates contemporary design concepts influenced by Transformers while maintaining the computational efficiency and inductive biases of convolutional networks. This approach provides better inference throughput while achieving accuracy comparable to Swin Transformer models at similar parameter scales, as reported in the original ConvNeXt study^15^. Because of these characteristics, ConvNeXt is especially well suited for STR, where the recognition process needs to work on structured text patterns while adhering to stringent latency and computational requirements. With an inference delay of 2.638 ms per image (NIVIDIA RTX 3050), the entire recognition pipeline in our implementation achieves 1.9 GFLOPs and 20.3 M parameters on a 32 × 100 input.

The ConvNeXt-based feature extractor converts raw pixel inputs into compact, high-dimensional feature representations that encode both semantic and spatial information relevant to scene text. These extracted features are designed to be robust to variations in font type, scale, and orientation, and resistant to background noise and visual clutter. Unlike conventional convolutional backbones, the proposed extractor incorporates a text-specific stem, multi-scale depth-wise convolutions, a multi-scale normalization scheme, and a tri-level attention mechanism (channel, spatial, and positional). Additionally, residual feed-forward blocks with layer-wise scaling and stochastic depth are employed to enhance representational diversity and stability. These components aim to (1) preserve fine-grained stroke and edge details, (2) capture long horizontal dependencies typical of text lines, and (3) suppress irrelevant background and channel noise.

This results in a compact and discriminative representation, denoted as F_final ∈ R^(B × 512 × 1 × 26) for input batches of size (  (B). Consequently, the feature extraction stage forms the core foundation of the STR pipeline, ensuring that rich and robust feature embeddings are delivered to the subsequent sequence modeling stage.

**A. Multi-Scale Layer Normalization Module**

**Fig. 4:**
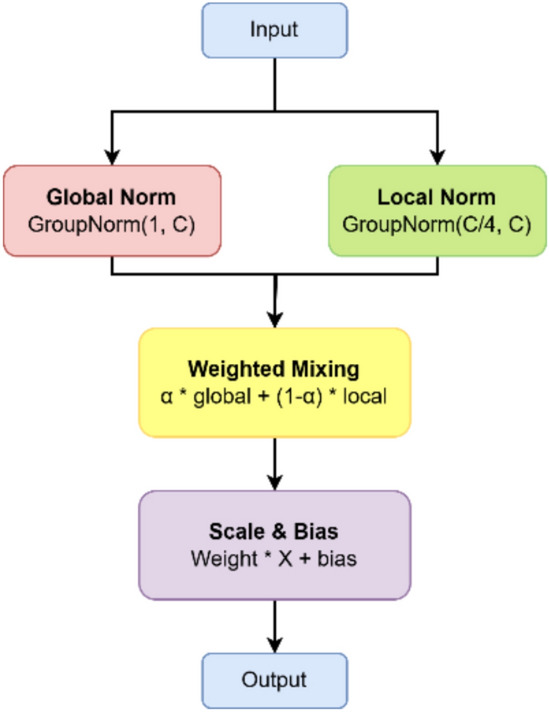
The architecture of multi-scale layer normalization.

As illustrated in Fig. 4, the Multi-Scale Layer Normalization (MSLN) module introduces a dual-path normalization strategy aimed at balancing both global and local statistical representations within the feature maps. Unlike conventional normalization methods that apply uniform scaling across all channels, the MSLN decomposes the normalization process into two complementary branches. One branch captures global contextual statistics, ensuring overall feature consistency, while the other focuses on local variations, preserving fine-grained structural details critical for text recognition.

This hybrid normalization mechanism enhances the model’s ability to maintain stability across varying spatial resolutions and complex background conditions, thereby improving the robustness and generalization of the extracted features.

In the first branch, global normalization is achieved using Group Normalization configured with a single group, effectively treating all feature channels as one collective unit. This approach ensures that normalization is applied across the entire feature map simultaneously, promoting global statistical consistency and stabilizing feature distributions throughout the network. By enforcing uniform scaling and centering across channels, the model captures a more holistic representation of the input x, which contributes to smoother optimization and improved generalization during training.

1 $${Norm}_{global}(x)=\frac{x-{\mu}_{g}}{\sqrt{{\sigma}_{g}^{2}+\epsilon }}$$

where ${\upmu}_{\mathrm{g}}$ and ${\upsigma}_{\mathrm{g}}^{2 }$ denote the global mean and variance across all channels, and ε is a very small constant.

In parallel, local normalization divides the feature channels into multiple smaller groups, allowing the network to retain fine-grained structural and contextual details within each subset. By computing normalization statistics (mean and variance) separately for each group, this path effectively captures intra-group dependencies, preserving subtle variations such as stroke thickness, edge patterns, and localized text features. This localized statistical modeling enhances the model’s ability to differentiate characters and word structures under varying fonts, scales, and distortions.

2 $${Norm}_{local}(x)=\frac{x-{\mu}_{l}}{\sqrt{{\sigma}_{l}^{2}+\epsilon }}$$

The final normalized representation is obtained by combining the outputs of both normalization pathways through a learnable weighted fusion mechanism, ensuring an optimal balance between global stability and local adaptability. Mathematically, it is defined as:

3 $$\widehat{X}= \alpha \cdot {Norm}_{global}\left(x\right)+(1-\alpha )\cdot {Norm}_{local}(x)$$

where $\alpha \in \left[\mathrm{0,1}\right]$ is a learnable parameter that adaptively controls the contribution of each normalization component during training. This formulation allows the network to dynamically emphasize either global coherence or local sensitivity based on the complexity and variability of the scene text.

**B. Text Attention Block (TAB)**

**Fig. 5:**
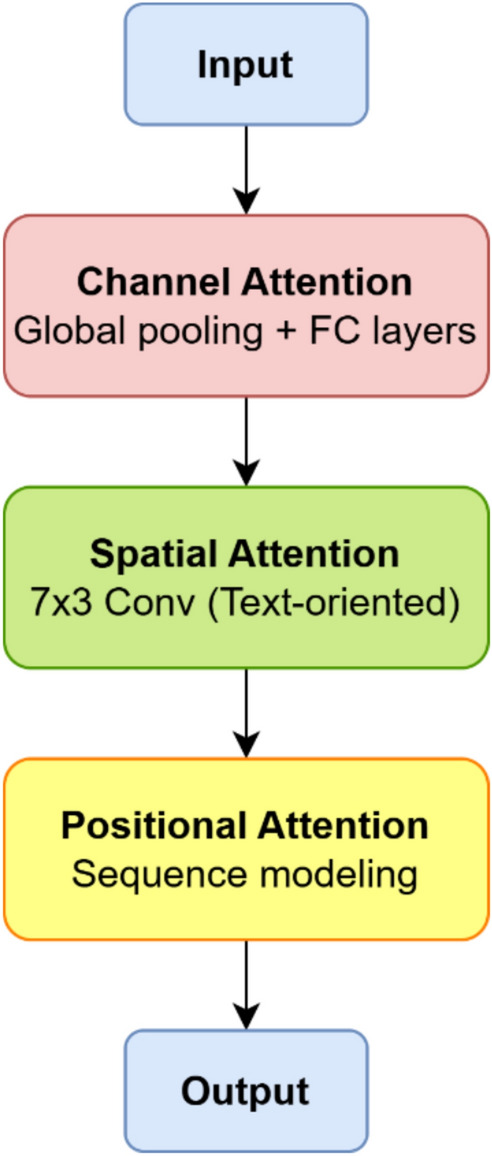
The architecture of the text attention block.

Enhances the discriminative capability of the extracted features by integrating three complementary attention mechanisms—channel, spatial, and positional—as illustrated in Fig. 5. Each mechanism focuses on a distinct aspect of the text representation to ensure more context-aware and semantically rich feature encoding. The channel attention emphasizes the most informative feature maps, the spatial attention highlights key text regions within the image, and the positional attention preserves the sequential ordering of characters, which is essential for accurate text recognition. Together, these mechanisms enable the network to effectively capture both local and global dependencies across irregular and cluttered scene text environments.

(1) Channel Attention learns inter-channel dependencies by modeling how different feature channels interact. Given a globally pooled descriptor z, channel attention weights are computed as:

4 $${s}_{c}=\sigma ({W}_{2}\cdot \delta ({W}_{1}z))$$

where ${W}_{1}$ and ${W}_{2}$ are trainable weight matrices, δ(⋅) denotes the ReLU activation function, and σ(⋅) represents the sigmoid function, and z = AdaptiveAvgPool2d(1)(x) ∈ ℝ^C is the globally average-pooled channel descriptor (global average pooling is used exclusively to capture smooth channel statistics robust to outlier activations in complex backgrounds). This formulation is structurally equivalent to the SE block; the novelty of TAB lies in the additional spatial and positional branches described below. By re-scaling channel responses according to ${s}_{c} .$ The module effectively amplifies discriminative features and suppresses redundant or noisy activations, thereby enhancing the representational capacity of the feature maps.

(2) Spatial attention captures spatial dependencies specific to text patterns, particularly the elongated horizontal structures commonly observed in scene text. It operates by aggregating both global average-pooled and max-pooled features, followed by a convolutional operation that emphasizes extended spatial regions.

The Spatial attention map is computed as:

5 $${S}_{s}=\sigma ({f}_{7\times 3}([AvgPool(x);MaxPool(x)]))$$

where ${f}_{7\times 3}$ denotes an asymmetric convolution kernel designed to prioritize horizontal continuity—a crucial characteristic for recognizing word-like sequences.

(3) Positional Attention incorporates sequential priority by applying lightweight convolutional transformations that encode positional dependencies within the feature maps’ inputs x:

6 $${s}_{p}=\sigma ({W}_{p}*x)$$

where ${W}_{p}$ prepresents a learnable convolutional kernel responsible for modeling positional context.

The final attended representation is then obtained through element-wise multiplication of the three attention maps:

7 $$x{\prime}=x\odot {s}_{c}\odot {s}_{s}\odot {s}_{p}$$

where ⊙ denotes element-wise multiplication. By jointly integrating channel, spatial, and positional cues, the module effectively emphasizes semantically relevant regions while suppressing background noise, thereby enhancing recognition robustness in complex scene text environments.

**C. Advanced Text Convolutional Block**

It serves as the fundamental computational unit within the proposed ConvNeXt-based backbone, designed to achieve an optimal balance between computational efficiency and representational richness. The block integrates multi-scale depth-wise convolutions, normalization, attention, and residual learning into a cohesive framework that enhances both local feature extraction and global contextual understanding.

(1) Multi-Scale Depth-wise Convolutions: To effectively capture diverse text patterns, the input channels are divided into multiple groups, each processed using depth-wise convolutions with distinct receptive fields—$(3\times 3),(5\times 3)$, and $(7\times 5)$. This multi-scale configuration allows the model to simultaneously encode fine-grained stroke details and broader contextual structures:

8 $${x}_{ms}={f}_{mix}\left(\left[{DW}_{3\times 3}\left({x}_{1}\right), {DW}_{5\times 3}\left({x}_{2}\right), {DW}_{7\times 5}\left({x}_{3}\right)\right]\right)$$

The 3 × 3 kernel captures stroke-level high-frequency details, including serifs and stroke terminals. The asymmetric 5 × 3 kernel captures character-scale features with horizontal emphasis matching the elongated structure of individual text characters. The 7 × 5 kernel captures word-level contextual patterns while retaining moderate vertical sensitivity. The asymmetric designs in the medium and large branches reflect the stronger horizontal than vertical structure of scene text, making isotropic kernels suboptimal for this task. The selected kernel configuration was adopted during model development as it provided a balanced receptive field for capturing both fine-grained character details and broader contextual patterns in scene text.

(2) Normalization and Attention: The fused feature maps are subsequently normalized using the proposed Multi-Scale Layer Normalization module and refined through the Text Attention Block, enabling adaptive feature reweighting and improved discriminative focus:

9 $$\begin{array}{cccc}& {x}_{att}=Attention(Norm({x}_{ms}))& & \end{array}$$

(3) Feed-Forward Network (FFN): A depth-wise separable feed-forward network is employed to enhance non-linear transformation and projection efficiency. It expands, filters, and projects the features through successive transformations:

10 $${x}_{ffn}={W}_{3}(\delta (DW({W}_{2}(\delta ({W}_{1}{x}_{att})))))$$

**Fig. 6:**
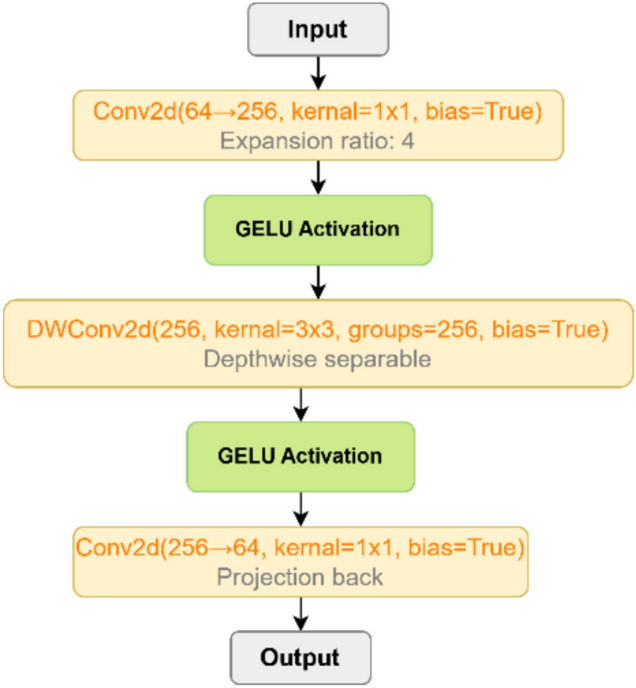
The architecture of a Feed Forward Network(FFN).

As illustrated in Fig. 6, this structure enhances the model’s capacity to learn hierarchical representations by combining channel expansion, spatial refinement, and efficient projection. The ATCB thus reinforces both local texture modeling and global semantic understanding—two critical aspects for achieving robust and accurate STR across complex visual environments.

(4) Residual and Stochastic Depth: To enhance training stability, the block incorporates residual learning combined with stochastic depth regularization:

11 $$y=x+\lambda \cdot DropPath({x}_{ffn})$$

Here, λ represents a learnable scaling parameter. The drop rate follows a linear schedule from 0.0 at the first ATCB block to 0.2 at the final block: drop_path_rates = linspace (0, 0.2, 11). This ensures minimal regularization of early feature layers and progressively stronger regularization of higher-level semantic layers. This design enables efficient training and effectively captures multi-scale and hierarchical textual representations.

(5) Text-Specific Stem module provides an enriched initialization for text recognition by utilizing four parallel convolutional branches, each designed to specialize in different types of textual features. The Fine Branch extracts high-frequency, stroke-level details essential for recognizing subtle character components. The Coarse Branch captures low-frequency, global character shapes that help in identifying overall letter structures. The Edge Branch focuses on boundary-sensitive features, which are crucial for distinguishing overlapping or closely spaced characters. Finally, the Context Branch utilizes elongated kernels to capture sequential dependencies across extended horizontal spans, thereby enabling the model to understand contextual relationships within words better. Together, these branches create a comprehensive and balanced feature representation at the earliest stage of processing. The outputs are concatenated and fused using a 3 × 3 convolution:

12 $${x}_{stem}={f}_{fusion}(\left[{x}_{fine},{x}_{coarse},{x}_{edge},{x}_{context}\right])$$

This fusion strategy ensures that both local precision and global structural information are preserved at the earliest stage of processing.

**D. SuperTextConvNext**

Backbone organizes the previously defined modules into a hierarchical pipeline specifically designed for text recognition. In Stage 1 (Fine Details), the network captures stroke-level information using shallow convolutional blocks and pooling operations to extract fundamental visual patterns. Stage 2 (Character-Level Processing) refines these extracted features for individual character representations through deeper stacked convolutional layers, enhancing the model’s ability to distinguish between similar characters. Moving to Stage 3 (Word-Level Context), the architecture introduces asymmetric pooling with a ratio of (2,1), which reduces the vertical resolution while maintaining horizontal continuity, an essential property for preserving word-level structures in text images. Finally, Stage 4 (Sequence Preparation) generates compact feature maps optimized for sequential decoders, ensuring that the output is well-suited for subsequent sequence modeling tasks such as text line recognition or transcription.

The final representation is a sequence-ready tensor:

13 $${F}_{final}=Pad\left(x,\left(1, 0, 0, 0\right)\right) \epsilon {\mathbb{R}}^{[B,\mathrm{512,1},{T}_{Seq}]}$$

Here, B denotes the batch size, and ${T}_{Seq}$ represents the sequence length. This padding operation ensures dimensional compatibility with sequence modeling layers such as BiLSTM or Transformer-based decoders, thereby enabling seamless integration of spatial feature maps into a sequential architecture for accurate and context-aware text recognition.

#### Sequence modeling

STR requires perceiving text as a continuous sequence of characters rather than as separate visual symbols. To effectively capture contextual dependencies among these sequential features, this stage employs advanced sequence modeling techniques. A BiLSTM network is commonly used for this purpose^67^, as it processes the extracted feature representations in both forward and backward directions. This bidirectional flow enables the model to gather contextual cues from the entire sequence, enhancing its understanding of character relationships within words. By modeling these sequential dependencies, the BiLSTM reduces ambiguity among visually similar characters and ensures that recognition decisions are guided not only by local visual details but also by the broader contextual information surrounding each character.

A standard LSTM cell updates its internal state according to:

14 $${f}_{t} = \sigma ({U}_{g}{x}_{t}+{W}_{g}{h}_{t-1}+{b}_{f})$$

15 $${i}_{t}=\sigma \left({U}_{i}{x}_{i}+{W}_{i}{h}_{t-1}+{b}_{i}\right)$$

16 $${\widetilde{m}}_{t}=tanh\left({U}_{m}{x}_{t}+{W}_{m}{h}_{t-1}+{b}_{m}\right)$$

17 $${m}_{t}=\left({g}_{t}*{m}_{t-1}+{i}_{t}*{\widetilde{m}}_{t}\right)$$

18 $${o}_{t}=\sigma \left({U}_{o}{x}_{t}+{W}_{g}{h}_{t-1}+{b}_{o}\right)$$

19 $${h}_{t}= {o}_{t}*\mathit{tan}h\left({c}_{t}\right)$$

where *U* and *W* are input weights in the gates of the input ( ${i}_{t}$), modulate input ( ${\widetilde{m}}_{t}$), forget (${f}_{t}$), and output (${o}_{t}$). ${h}_{t}$, ${c}_{t}$, and *b* are a hidden condition, cell state, and bias function, respectively.

In the bidirectional configuration, two independent LSTM layers are employed to capture contextual dependencies in both temporal directions. Specifically, the forward layer processes the sequence from left to right, yielding $(\overrightarrow{{h}_{t}})$, while the backward layer processes the same sequence from right to left, producing $({\overleftarrow{h}}_{t})$.

The two hidden states are then concatenated to form a comprehensive representation at each time step:

20 $${H}_{t}=\left[\overrightarrow{{h}_{t}};{\overleftarrow{h}}_{t}\right]$$

When applied to the fused feature sequence ${F}_{final}$, the bidirectional LSTM operation can be formalized as:

21 $${H}_{t}=BiLSTM({F}_{final})$$

${H}_{t}$ denotes the contextually enriched feature vector at time step t, encoding information from both preceding and succeeding elements in the sequence. This bidirectional modeling enhances the representation of the spatially extracted features by incorporating long-range dependencies in both directions. Consequently, the output sequence provides a semantically rich representation that is particularly well-suited for downstream tasks such as transcription and attention-based decoding.

#### Prediction stage

At the prediction stage, an attention-based mechanism is employed as the core component for generating the output text sequence. The decoder functions in an autoregressive fashion, producing one character at a time while conditioning each prediction on both the previously generated symbols and the encoded visual features. This architecture allows the model to effectively learn character-level language dependencies and dynamically capture contextual relationships between characters, which is especially beneficial in handling challenging cases such as irregular text shapes, curved baselines, or noisy backgrounds. Consequently, the attention mechanism enhances the model’s ability to produce robust and accurate transcriptions. Formally, the attention weight assigned to the ${i}^{th}$ encoder output at decoding step (t) is calculated as:

22 $${\alpha}_{t,i}=\frac{\mathit{exp}\left( {e}_{t,i}\right)}{{\sum}_{k=1}^{n}{e}_{t,k}}, {e}_{t,i}={v}_{a}^{T}tanh\left({W}_{s}{S}_{t-1}+{W}_{h}{H}_{i}\right)$$

where ${S}_{t-1}$ denotes the decoder state at the previous step, ${H}_{i}$ represents the encoder hidden vector at position *i*, ${W}_{s}$ and ${W}_{h}$ are learnable projection matrices, and ${V}_{a}$ is a trainable vector parameterizing the alignment function.

The context vector is then derived as a weighted sum of the encoder hidden states:

23 $${c}_{t}=\sum_{i=1}^{n}{\alpha}_{t,i}{H}_{i}$$

The decoder subsequently generates the probability distribution over the character vocabulary as:

24 $${y}_{t}=softmax({W}_{o}\left[{S}_{t};{C}_{t}\right])$$

where ${W}_{o}$ is the output projection matrix, and $\left[{S}_{t}; {C}_{t}\right]$ denotes the concatenation of the decoder state and context vector.

Finally, the network outputs the complete transcription sequence as an ordered set of predicted characters:

25 $$Y={(y}_{1}, {y}_{2}, \dots .., {y}_{T})$$

#### Loss function

To optimize the training process and effectively handle both class imbalance and model overconfidence, a combined loss function strategy is employed—utilizing Focal Loss during synthetic pre-training and Label Smoothing Loss during fine-tuning on real-world datasets.

**A. Focal Loss**

Focal Loss is introduced to alleviate the problem of class imbalance, which commonly occurs in large-scale synthetic datasets where certain characters or symbols appear more frequently than others. Its formulation is defined as:

26 $$FL({p}_{t})=-\alpha {(1-{p}_{t})}^{\gamma }log({p}_{t})$$

where ${p}_{t}$ represents the predicted probability corresponding to the ground-truth class, α is a balancing factor that adjusts the importance of positive and negative samples, and γ is a focusing parameter that reduces the loss contribution from well-classified examples. This mechanism directs the model’s attention toward harder or misclassified samples, enhancing its robustness and improving recognition accuracy for complex, distorted, or irregular text patterns.

**B. Label Smoothing**

To further enhance generalization and mitigate overconfidence in model predictions, Label Smoothing Regularization (LSR) is employed. Rather than training the network using strict one-hot ground-truth labels, the target distribution is slightly softened to prevent the model from assigning full probability mass to a single class. The smoothed target distribution is defined as:

27 $${y}_{i}^{smooth}=(1-\epsilon )\cdot {y}_{i}^{onehot}+\frac{\epsilon }{K}$$

where K is the number of classes and ϵ is the smoothing parameter. During pre-training on synthetic datasets, ϵ = 0.1 is used to prevent the model from overfitting the large but potentially noisy dataset. In the fine-tuning stage on real-world datasets, a smaller value of ϵ = 0.05 is adopted, allowing the network to place higher confidence on the ground-truth labels while still maintaining robustness to annotation noise.

**C. Combined Loss Strategy**

To enhance training efficiency and balance robustness with generalization, a combined loss strategy is adopted across different training phases. During synthetic pre-training, the model utilizes a weighted combination of Focal Loss and Label Smoothing Loss, formulated as:

28 $${L}_{pretrain}=0.7\cdot {L}_{Focal}+0.3\cdot {L}_{LS}$$

This formulation allows the network to leverage the hard sample focusing capability of Focal Loss while simultaneously benefiting from the regularization and generalization effects of Label Smoothing. The 0.7/0.3 weighting was determined through preliminary experiments during model development, reflecting the relative importance of class imbalance correction (Focal Loss) versus overconfidence regularization (Label Smoothing) for large-scale synthetic training data.

During fine-tuning on real-world datasets, the objective function is simplified to rely solely on Label Smoothing Loss, expressed as:

29 $${L}_{finetune}={L}_{LS} (\epsilon =0.05)$$

This adjustment reflects the smaller and more reliable nature of real datasets, where the key challenge lies in reducing overconfidence rather than addressing class imbalance. As a result, this two-stage loss design ensures stable convergence during pre-training and improved generalization during fine-tuning.

## Results and discussion

### Implementation details

This section outlines the experimental setup, training strategy, and evaluation procedure adopted to assess the proposed ConvNext Feature Extraction Model for STR.

#### Model and training strategy

The proposed approach employs a ConvNext-based feature extraction backbone, designed to efficiently capture multi-scale text patterns and enhance representational power while preserving computational efficiency. ConvNext integrates depthwise convolutions, inverted bottlenecks, and advanced normalization schemes, which collectively enable the model to learn robust hierarchical features suitable for irregular, multi-lingual, and noisy text images. To address class imbalance across datasets, equal-sized samples were selected to construct balanced mini-batches, ensuring more stable training and improved generalization across diverse text distributions.

The training process was conducted in two sequential stages:

Pre-training on Synthetic Datasets: The model was initially trained using large-scale synthetic datasets, MJSynth and SynthText, which provide a wide variety of fonts, styles, distortions, and orientations. This stage facilitated robust feature learning, allowing the ConvNext backbone to capture both local structural cues (e.g., strokes and edges) and global spatial patterns. Training was performed with the AdamW optimizer, an initial learning rate of 0.001, a OneCycle learning rate scheduler, and a combined loss strategy defined as:

$${L}_{pretrain}=0.7\cdot {L}_{Focal }\left(\gamma =2.0,\alpha =1.0\right)+ 0.3\cdot {L}_{LS}\left(\epsilon =0.1\right).$$

This configuration allowed the model to emphasize hard-to-classify samples through Focal Loss while Label Smoothing reduced overconfidence and improved generalization under noisy synthetic conditions.

Fine-tuning on Real-world Datasets: After pre-training, the model was fine-tuned on a union of real-world datasets, including IC13, IC15, RCTW, ArT, LSVT, MLT19, ReCTS, COCO-Text, Uber-Text, TextOCR, OpenVINO, and a subset of Union14M-L. Fine-tuning was performed with a reduced learning rate of 0.0001, and Label Smoothing with a reduced smoothing factor defined as:

$${L}_{finetune}={L}_{LS} (\epsilon =0.05)$$

This adjustment enabled the model to place higher confidence on reliable real-world labels while maintaining resilience against annotation noise and allowing the model to adapt to the complexities of natural scene texts, such as background clutter, non-uniform illumination, and irregular orientations. This stage enhanced robustness and improved recognition accuracy under real-world conditions.

The proposed design aims to strike a balance between feature expressiveness and generalization capacity while minimizing the risk of overfitting. Following extensive ablation studies, the selected training configuration demonstrated consistent improvements. Model selection was based on performance on the validation set, with evaluations conducted every 2,000 iterations. The best-performing model on the validation set was preserved for final testing, thereby confirming its ability to generalize effectively to unseen datasets. Table 5 presents the hyperparameter settings of the proposed model, and Table 6 summarizes the computational efficiency of the complete recognition pipeline, while Table 7 summarizes the system configuration used for model implementation.

**Table 5:** Hyperparameter settings in the proposed system.

Parameter	Value
Optimizer	AdamW
Learning_rate	0.001 (initial), 0.0001 (fine-tuning)
Scheduler	OneCycle
Batch_size	128
Balancing factor (α)	1.0
Focusing parameter (γ)	2.0
Smoothing parameter (ε)	0.1 (initial), 0.05 (fine-tuning)

**Table 6:** Computational efficiency of the proposed model.

Metric	Value
N of Parameters	20.3 M
GFLOPs	1.9
Model size	78.1 MB
Inference latency	2.638 ms per image

**Table 7:** System configuration for model implementation.

System Description	Details
Programming language	Python
Operating system	Windows 10
Processor	12th Gen Intel®Core™ i5-12500H, 2.5 GHz, 12 Cores, 16 Threads
GPU	NVIDIA GeForce RTX 3050
RAM	8 GB

The computational efficiency of the complete recognition pipeline (TPS + ConvNext + BiLSTM + Attention Decoder) was measured on a single input of size 32 × 100. On a single NVIDIA GeForce RTX 3050 GPU, the model achieves 20.3 M parameters, 1.9 GFLOPs, a model size of 78.01 MB, and an inference latency of 2.638 ms per image. These findings show that the suggested architecture strikes a good compromise between computing economy and recognition accuracy, making it appropriate for real-time applications and possible implementation in settings with limited resources.

#### Evaluation metric

The model’s performance was assessed using word-level accuracy, focusing on alphanumeric characters, as is standard in STR benchmarks^68^. Six benchmark datasets were used to calculate the total accuracy, which is defined as the cumulative accuracy across all datasets. All experiments were repeated twice with different random initializations, and the average accuracy was reported to ensure consistency and reproducibility of the results.

30 $${\boldsymbol{Accuracy}}=\frac{{\boldsymbol{No~ of~ correctly ~predicted~ words}}}{{\boldsymbol{Total ~No~ of ~words~ in~ the~ dataset}}}\times 100$$

31 $${\boldsymbol{Nor}}{{\boldsymbol{m}}}_{{\boldsymbol{ED}}}=\frac{1}{{\boldsymbol{N}}}\sum_{{\boldsymbol{i}}=1}^{\_{\boldsymbol{N}}}\left(1-\frac{{\boldsymbol{Edit ~Distance}}({pred}_{i },{gt}_{i})}{{\boldsymbol{Max}}({\boldsymbol{len}}\left({gt}_{i}\right),{\boldsymbol{len}}\left({pred}_{i }\right))}\right)$$

where N is the total number of text samples in the dataset, ${pred}_{i}$ is the predicted text for the ${\mathrm{i}}^{\mathrm{th}}$ sample, ${gt}_{i}$ is the ground truth text for the ${\mathrm{i}}^{\mathrm{th}}$ sample, Edit Distance( ${pred}_{i}$, ${gt}_{i}$), is the Levenshtein distance (character-level difference) and $\mathrm{Max}(\mathrm{len}\left({gt}_{i}\right), \mathrm{len}\left({pred}_{i }\right))$ is used for normalization.

The recognition system recognizes 36 characters, including lowercase English letters and numerals 0–9. During evaluation, accuracy is measured by the number of properly predicted words, and the normalized edit distance (NED) analyzes character-level similarity between the predicted and ground truth words.

### Comparison of state-of-the-art methods

Table 8 compares the performance of state-of-the-art approaches to our proposed ConvNext Model, and Table 9 reports the Normalized Edit Distance (NED) of the Proposed Model on Standard Benchmarks. To guarantee a fair evaluation, we compared models trained and evaluated on six different benchmark datasets: IIIT, SVT, IC13-1015, IC15-2077, SP, and CT. The baseline model, as reported in previous investigations, has an accuracy of 82.9%.

**Table 8:** Comparisons of STR performance with previous methods on several benchmarks.

Dataset	IIIT5k	SVT	IC13-857	IC13-1015	IC15-1811	IC15-2077	SVTP	CUTE80
NED	0.991	0.990	0.995	0.992	0.969	0.967	0.978	0.988

**Table 9:** Normalized edit distance (NED) of the proposed model on standard benchmarks.

Dataset	IIIT5k	SVT	IC13-857	IC13-1015	IC15-1811	IC15-2077	SVTP	CUTE80
NED	0.991	0.990	0.995	0.992	0.969	0.967	0.978	0.988

NED values confirm consistent character-level recognition quality across both regular and irregular benchmarks, with all values exceeding 0.96.

Our ConvNeXt-based STR model demonstrated notable improvements in recognition performance across both synthetic and real-world datasets. When trained exclusively on synthetic data—specifically the MJSynth dataset (9 million samples) and SynthText dataset (7 million samples) the model achieved an accuracy of 89.1%, marking a 6.2% improvement over the baseline model under identical training conditions. After fine-tuning on a comprehensive set of real-world datasets, including IC13, IC15, RCTW, ArT, LSVT, MLT19, ReCTS, COCO-Text, Uber-Text, TextOCR, OpenVINO, and a subset of Union14M-L, the accuracy further increased to 94.71%, representing an 11.81% gain compared to the baseline trained on both synthetic and real data. A further experiment was carried out in which the model was trained solely on real-world datasets, without any synthetic pre-training, in order to further separate the contributions of the suggested architecture and the training data regime. Under these circumstances, the model achieves an average accuracy of 94.25%, which is only 0.46% lower than the full synthetic + real result of 94.71%. This indicates that the architectural design is the main factor influencing recognition performance, with synthetic pre-training offering a slight supplementary benefit through additional font and style diversity.

The superior performance of the ConvNeXt-based framework stems from its modern architectural innovations, which include depth-wise convolutions, inverted bottleneck modules, and multi-scale normalization layers. These components work synergistically to enhance feature discrimination, allowing the model to effectively capture both fine-grained text details and global contextual dependencies. Additionally, the two-phase training paradigm—synthetic pre-training followed by real-world fine-tuning—significantly boosts generalization, reducing susceptibility to background noise, irregular text orientations, and multilingual variations.

Overall, these results underscore the efficiency and robustness of the ConvNeXt-based architecture in tackling the challenges inherent to STR, offering a streamlined yet powerful alternative to traditional hybrid feature fusion approaches.

### Ablation study

To rigorously validate the effectiveness of each proposed architectural component, we conduct a systematic ablation study. Starting from a baseline configuration, we incrementally add each module and measure its contribution to recognition performance. All experiments are performed using identical training conditions to isolate the impact of architectural modifications.

#### Experimental setup

Our baseline model consists of TPS transformation, standard ConvNeXt backbone with uniform 7 × 7 depthwise convolutions and conventional Layer Normalization, BiLSTM sequence modeling, and attention-based prediction. To ensure fair and reproducible comparison, all ablation experiments follow strictly controlled conditions: Initialization: All configurations start from the same random initialization (seed equals 1111), ensuring that performance differences reflect architectural improvements rather than initialization variance. Training Data: All models are trained on real-world data consisting of the union of 12 benchmark datasets (IC13, IC15, RCTW, ArT, LSVT, MLT19, ReCTS, COCO-Text, Uber-Text, TextOCR, OpenVINO, and a Union14M-L subset). This approach directly evaluates architectural effectiveness on realistic, diverse text samples rather than synthetic data. Training Duration: Each configuration is trained for 3 epochs, which provides sufficient convergence while maintaining computational feasibility for multiple ablation runs.

To guarantee consistency across all ablation settings, identical hyperparameters are employed throughout the experiments. The models are optimized using AdamW with a weight decay of 0.05 and an initial learning rate of 1 × 10^–4^, scheduled using the OneCycle policy. A batch size of 64 is used, and label smoothing with a smoothing factor ϵ = 0.05 is applied to stabilize training. All experiments are conducted on a single NVIDIA GeForce RTX 3050 GPU, and the input images are resized to a fixed resolution of 32 × 100pixels.

Evaluation Protocol: Each configuration is evaluated on six standard benchmark datasets: IIIT5k, SVT, IC13, IC15, SVTP, and CUTE80. All evaluations follow a case-insensitive, lexicon-free word-level accuracy measurement. Due to identical initialization and deterministic training, results are highly reproducible with negligible variance across repeated runs.

#### Component-wise results

Table 10 presents the systematic ablation results with per-dataset accuracy and grouped averages for regular and irregular benchmarks. The per-category breakdown reveals that each proposed component addresses distinct challenges within the recognition pipeline.

**Table 10:** Ablation Study: Incremental Component Contribution. Avg Reg represents average accuracy for the regular dataset over IIIT5k, SVT, IC13-857, and IC13-1015. Avg Irreg represents average accuracy for the irregular dataset over IC15-1811, IC15-2077, SVTP, and CUTE80. Acc represents average accuracy over all six benchmarks. Δ represents improvement over the immediately previous configuration.

Configuration	Regular				Avg Reg	Irregular				Avg Irreg	Acc	Δ (%)
	IIIT 3000	SVT 647	IC13			IC15		SVTP 645	CUTE 288			
			857	1015		1811	2077					
Baseline (Basic TPS + ConvNext + BiLSTM)	92.63	88.87	94.05	94.19	92.7	84.04	83.77	82.64	86.41	83.88	88.7	-
Add MultiScaleLayerNorm2d	92.53	90.26	94.05	94.29	92.82	84.65	84.35	84.81	86.76	84.67	89.1	+ 0.4
Add Text Specific Stem	92.6	90.73	94.28	94.38	92.97	84.98	84.65	84.5	89.55	85.05	89.3	+ 0.2
Add Text Attention Block	93.23	90.57	93.7	93.89	93.11	85.31	84.96	83.10	86.76	84.95	89.4	+ 0.1
Full Model (All components)	92.9	90.88	95.1	95.27	93.44	85.09	84.76	84.03	88.15	84.99	89.6	+ 0.2
Full Model without TPS	92.4	90.42	94.4	94.38	92.84	83.10	80.75	81.09	86.76	82.04	87.9	-1.7

Δ represents improvement over the immediately previous configuration. All models were trained on real-world data with identical initialization (seed = 1111) and hyperparameters for fair comparison.

MSLN yields a substantially larger improvement on irregular text (+ 0.79%) than on regular text (+ 0.12%), confirming that adaptive normalization between global and local statistics is particularly effective for handling the diverse appearance variations—including curved baselines, non-uniform illumination, and complex backgrounds—that characterize irregular text instances.

Text Specific Stem contributes positively to both categories (+ 0.15% regular, + 0.38% irregular), with a stronger effect on irregular text, consistent with its multi-branch design capturing features at multiple scales and orientations.

TAB improves regular text recognition (+ 0.14%) through its channel and spatial attention mechanisms, with a marginal trade-off on irregular text (− 0.10%). This reflects the complementary nature of the attention components: the channel and spatial branches primarily refine structured horizontal text patterns, while the positional branch targets sequential ordering—an interaction whose combined effect on highly irregular samples is modulated by the preceding Text Specific Stem features.

Finally, removing TPS causes a substantially larger degradation on irregular text (− 2.95%) than on regular text (− 0.60%), confirming that spatial rectification is a non-redundant component whose primary role is geometric normalization of distorted text instances.

### Discussion

The experimental results allow for the separation of the relative contributions of training strategy and architectural innovation. When the architectural contribution is isolated under set training conditions, the ablation research (Table 10) consistently yields a + 0.9% gain from Baseline to Full Model. The synthetic-only to synthetic + real gap, which is + 5.61% under fixed architecture, serves as a measure of the training strategy’s contribution. Crucially, the real-data-only experiment (94.25%) shows that the suggested architecture achieves near-peak performance without synthetic pre-training—just 0.46% below the complete result—confirming that architectural design is the main factor influencing recognition performance, with the two-stage training approach offering complementary benefits for generalization.

- Failure Case Analysis

**Fig. 7:**
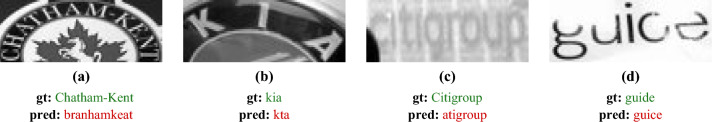
Representative failure cases of the proposed model: (**a**) severe blur and circular distortion, (**b**) low contrast and specular, (**c**) non-uniform, (**d**) artistic font.

Figure 7 presents four representative failure cases drawn from the evaluation benchmarks, covering the main failure categories of the proposed model. gt stands for ground truth, and pred stands for prediction.

Four typical failure cases from each of the primary failure categories of the suggested model are shown in Fig. 7. (a) Severe blur and circular distortion: Because TPS rectification is unable to recover a horizontal baseline from circular text layouts, circular logo text with extreme blur and perspective distortion results in widespread character misrecognition. (b) Low contrast and specular reflection: non-uniform local contrast produced by metallic surface reflections suppresses stroke feature responses and results in character deletion. (c) Non-uniform illumination: despite MSLN normalization, localized low contrast brought on by transparency or faded printing considerably reduces the first character’s feature response. (d) Artistic font: Because the artistic font significantly departs from the training distribution, highly stylized letterforms lead to character misunderstanding (d → c). Future research on illumination-robust preprocessing, improved rectification for non-horizontal text layouts, and augmentation with a variety of creative font styles is motivated by these failure mechanisms.

- Limitations:

First, the current framework is primarily made for text with a horizontal structure since both the TPS rectification and the asymmetric convolutional kernels emphasize horizontal elements. When dealing with vertically oriented text, which often happens in some real-world scenarios like East Asian signage, additional rotation-aware preprocessing or modified rectification procedures may be useful.

Second, very small words occupying vast image regions may result in very sparse feature representations, whereas very long words with many characters may experience compression effects due to the fixed 32 × 100 input resolution. As a potential future enhancement, adaptive resolution scaling might be investigated.

Finally, recognition performance may still be adversely affected by very severe lighting fluctuations or large shadow occlusions due to lower local contrast, even though training with real-world datasets affords some robustness to illumination variation. Using more advanced lighting normalization or augmentation techniques could increase robustness in such circumstances.

## Conclusion

STR has traditionally relied on large-scale synthetic datasets for model training, with real labeled data used sparingly due to their limited availability. In this work, we address this limitation by incorporating newly available large-scale real-world labeled datasets into the training pipeline and by introducing a ConvNeXt-based feature extraction architecture. The ConvNeXt backbone provides advanced convolutional representations inspired by transformer design principles, offering both local structural sensitivity and global contextual awareness. This allows the model to effectively handle diverse STR challenges such as irregular layouts, curved text, multilingual scripts, and complex real-world backgrounds.

The experimental findings clearly demonstrate the benefits of combining synthetic and real-world data in a two-stage training process. Pre-training on massive synthetic datasets such as MJSynth and SynthText enables the ConvNeXt backbone to capture a wide variety of font styles, distortions, and geometric patterns, thus establishing a strong foundation. Fine-tuning on real-world datasets further enhances the model’s robustness by exposing it to authentic variations, including background clutter, non-uniform illumination, and perspective distortions. As a result, the proposed ConvNeXt model achieved an average accuracy of 94.71% across multiple benchmarks, a significant improvement over the 89.1% accuracy obtained when trained solely on synthetic data.

These results highlight that architectural advancement and dataset diversity are critical to improving STR performance. The ConvNeXt-based design provides discriminative and semantically rich feature representations, while the balanced integration of synthetic and real data ensures strong generalization across unseen conditions. Overall, this work demonstrates that coupling modern feature extraction architectures like ConvNeXt with a diverse data regime is a powerful strategy for building adaptive, robust, and accurate STR systems. It also lays the groundwork for future research aimed at extending such architectures to increasingly complex real-world recognition scenarios. A promising direction is the incorporation of adversarial training principles and perturbation defense mechanisms^12^ into the STR training pipeline—for example, through adversarial sample augmentation to further improve model robustness against malicious noise, extreme geometric distortions, and other challenging input-level perturbations. Additionally, although the current framework only addresses Latin-script alphanumeric recognition, expanding the suggested architecture to non-Latin scripts—by modifying the vocabulary, prediction head, and training data—represents a significant and promising avenue for future research.

## Data Availability

All datasets used in this study are publicly available datasets commonly employed in scene text recognition research. No new datasets were generated or collected during the current study. Dataset access information:
